# The Impact of Waitlisting After a Weekend on Transplant‐Related Outcomes for Patients With Acute Liver Failure in the US

**DOI:** 10.1002/jgh3.70271

**Published:** 2025-09-01

**Authors:** Melis G. Celdir, Qianyi Shi, Tomohiro Tanaka

**Affiliations:** ^1^ Division of Gastroenterology Washington University School of Medicine St. Louis Missouri USA; ^2^ Department of Internal Medicine University of Iowa Carver College of Medicine Iowa City Iowa USA; ^3^ Division of Gastroenterology and Hepatology University of Iowa Carver College of Medicine Iowa City Iowa USA; ^4^ Department of Health Management and Policy, College of Public Health University of Iowa Iowa City Iowa USA

**Keywords:** acute liver failure, United Network for Organ Sharing, waitlist management

## Abstract

**Introduction:**

Potential delays in patient care during weekends have not been studied in liver transplantation (LT) for acute liver failure (ALF). We evaluated the impact of listing after a weekend on waitlist (WL) and post‐LT outcomes in ALF patients.

**Methods:**

In a retrospective cohort study of adult ALF patients from February 2002 to May 2023 in the United Network for Organ Sharing (UNOS) database, the primary exposure was listing after a weekend. Multinomial regression and Cox models assessed WL outcomes and post‐LT mortality, respectively, adjusting for potential confounders. Inverse probability censoring weighting addressed censoring bias.

**Results:**

Among 6600 adults listed for LT, 840 (13%) were listed after a weekend. The median number of days from admission to waitlisting was 2 (IQR1–3). Accounting for potential confounders, patients listed after a weekend had a lower likelihood of spontaneous survival (SS; relative risk ratio 0.74, 95% CI 0.61–0.90, LT as reference). Post‐LT mortality at 1 year was higher in patients listed after a weekend (HR 1.25, 95% CI: 1.01–1.54). IPW cohort outcomes corroborated these findings.

**Conclusions:**

Among adults with acute liver failure, listing for liver transplantation after a weekend was associated with lower rates of spontaneous survival and higher one‐year post‐transplant mortality. Identifying system‐level factors contributing to delays in transplant evaluation over weekends may help improve the timeliness and efficiency of care.

**Abbreviations**
AC: acuity circle
ALF: acute liver failure
CI: confidence interval
HR: hazard ratio
IPW: inverse probability weighting
LT: liver transplant
RRR: relative risk ratio
SS: spontaneous survival
UNOS: United Network for Organ Sharing
WL: waitlist

## 1 Introduction

Numerous studies on emergency medical and surgical conditions, including acute liver failure, suggest adverse outcomes associated with weekend hospitalizations [1, 2, 3, 4], whereas studies on the “weekend effect” in kidney and liver transplantation have yielded inconsistent results [5, 6, 7]. Acute clinical presentations with high immediate mortality linked to the timeliness of treatment are likely more prone to adverse outcomes associated with delays in care on weekends [8].

Acute liver failure (ALF) is a life‐threatening emergency requiring prompt coordination of complex care involving multidisciplinary teams [9, 10]. Up to 60% of listed patients receive liver transplant (LT) [11], and LT‐free mortality can be as high as 50% [12]. Although spontaneous survival (SS) rates have improved with advances in critical care, the overall rate remains at only 20%, and LT is the only definitive treatment for ALF [10]. Therefore, ALF patients who require LT evaluation on weekends are at high risk for adverse clinical outcomes due to delays in care. Prolonged time to LT listing can result in inferior waitlist (WL) and post‐LT outcomes. In a study using the UNOS database, the decline rate for adult livers was highest on the weekend, suggesting a weekend effect on liver offers [13]. While previous studies have explored various aspects of timing in transplantation, including weekend effects on organ acceptance and utilization [14], the specific impact of delayed listing after weekends on ALF outcomes has not been previously investigated. Prolonged time to LT listing can result in inferior waitlist (WL) and post‐LT outcomes. To our knowledge, the weekend effect in ALF has not been studied.

Using the United Network for Organ Sharing (UNOS) database, we assessed the impact of delays in care and the listing process over a weekend, with LT listing after a weekend serving as a proxy, on clinical outcomes in adult ALF patients in the United States. First, we evaluated the association of listing after a weekend on WL outcomes among all LT candidates with ALF. Second, we examined whether listing for ALF after a weekend is associated with higher post‐LT all‐cause mortality among those who underwent LT.

## 2 Materials and Methods

We performed a retrospective cohort study using the Organ Procurement and Transplantation Network (OPTN) UNOS Standard Transplant Analysis and Research (STAR) file, following the STROBE (Strengthening the Reporting of Observational Studies in Epidemiology) guidelines for reporting observational studies [15]. The content of the analysis is the responsibility of the authors alone and does not necessarily reflect the views or policies of the US Department of Health and Human Services. Mention of trade names, commercial products, or organizations does not imply endorsement by the US Government. This study was approved by the University of Iowa Institutional Review Board (No. 202305203).

### 2.1 Study Population

The cohort included all adults (age ≥ 18) with ALF who were placed on LT waitlist as status 1A from February 27, 2002 (MELD implementation date) to May 30, 2023, in the US, patients were followed until June 30, 2023. Only patients listed for LT alone were included in the study. Re‐transplantation episodes and observations without a specified waitlist outcome were excluded. Those waitlisted for more than 14 days were excluded specifically to avoid potential misclassification of ALF cases, given the anticipated short turnover. Observations with a waitlist date erroneously assigned earlier than the admission date in the dataset were found to be re‐transplantation episodes based on discharge diagnoses, such as graft failure or hepatic artery thrombosis. These observations were excluded. Figure S1 presents inclusion and exclusion criteria.

The etiology of ALF was determined based on coding and free text entry in the UNOS database at listing [16]. If an entry contained terms with a specific designation for acetaminophen toxicity such as “acetaminophen,” “Tylenol,” or “APAP,” ALF etiology was determined to be acetaminophen toxicity; if an etiology had other designations or was not specified, it was coded as “other.”

### 2.2 Exposure Variable and Outcomes

The primary exposure of interest was listing on the first day after a weekend. For weekends followed by a US federal Monday holiday (i.e., Martin Luther King Jr. Day, Memorial Day, Labor Day, New Year's Day [if Sunday or Monday], Independence Day [if Sunday or Monday], Christmas Day [if Sunday or Monday]), Tuesday was used. Therefore, the independent variable, *listing after a weekend*, was defined as non‐Holiday Mondays after a weekend and Tuesdays following a Monday‐holiday weekend. The values of all demographic and clinical variables included in this study were determined at the time of listing and included age, sex, race, ALF etiology, primary payer (Medicaid vs. others), and the era (before and after acuity circle [AC] policy implementation [2/4/2020]). Additional descriptive variables included MELD score, number of days on WL, and cold ischemia time.

Primary study outcomes for the first aim were receipt of LT, removal from WL due to death or being too sick for LT (WL mortality), and removal from WL due to clinical improvement/spontaneous survival. The outcome for the second aim was all‐cause mortality at 1 year after LT among patients who underwent LT (time zero = the day of LT).

### 2.3 Statistical Analyses

The statistical analyses were performed using R software version 4.4.0 (R Foundation for Statistical Computing, Vienna, Austria). The magnitude of missing data in the propensity score‐matched cohort was minimal (< 1% among covariates).

Baseline demographic and clinical characteristics of patients were summarized with frequency and percentages, median or means. The variables were compared between groups listed after a weekend and other days using chi‐square test and *t*‐test when applicable.

Given the short duration between listing and WL outcomes for LT candidates with ALF, a multinomial regression analysis was performed to evaluate the effect of listing after weekends on WL outcomes (LT, SS, WL mortality) [16, 17]. Models were adjusted for potential confounding variables: age, sex, race/ethnicity, ALF etiology (acetaminophen toxicity vs. others), before vs. after AC policy implementation, and Medicaid coverage (vs. other primary payers). We estimated adjusted predicted relative risk ratios (RRR) for SS or WL mortality with LT as a reference outcome.

To assess the effect of LT listing practices on post‐LT mortality for patients who underwent LT, we performed Cox proportional hazards models adjusted for the same variables used in the aforementioned multinomial models.

A direct acyclic graph (DAG) describing the causal relationship between WL practices and clinical outcomes is demonstrated in Figure S2. Listing after a weekend was considered a *measured* indicator of potential delays in listing sick patients who would have been listed for LT earlier in the week or during the weekend. Our DAG illustrated that a true delay in listing for LT was not measured but represented in the dataset as listing after a weekend, which is the measured value. Even if a patient was listed after a weekend but in a timely manner, this would be considered a *misclassified* categorical variable with regard to true delays in being listed for LT. However, we assumed this misclassification to be non‐differential because the factors responsible for this misclassification of the exposure variable “delays in listing” over a weekend are independent of the factors affecting the outcome measures [18, 19]. For binary mismeasured variables, risk estimates for the mismeasured variable lie between the crude and true measures i.e., non‐differential misclassification typically results in a bias toward the null [19].

In post‐LT analyses, we assumed that a selection bias was introduced to the analytic cohort that included only patients who underwent LT. The WL outcomes of SS or death without LT were not observed but would have affected posttransplant outcomes had they occurred. This censoring bias due to including only patients who received LT was addressed using inverse probability weighting (IPW) [20]. Two separate analyses were performed to answer our causal questions: (1) what if the patients who were not transplanted and died (WL mortality) would have been transplanted, and (2) what if the patients who survived without LT (SS) would have been transplanted? The probability that a patient drops out of the WL due to (1) WL mortality and (2) SS was estimated in two separate models using logistic regression that included the following variables: sex, age at WL, ALF etiology, pre–post‐AC era, insurance, primary payer (Medicaid vs. others), race/ethnicity, and transplant center volume. Of note, variables lying on the causal pathway, such as MELD score, hospitalization/ICU status, hemodialysis, vasopressor use, functional status, and hepatic decompensation (e.g., hepatic encephalopathy and ascites), were purposefully excluded from these models to avoid overadjustment; see Figure S2 for the DAG [21, 22]. The resulting probabilities formed the basis for the propensity scores used to generate IPCW.

We performed two additional sensitivity analyses: (1) we repeated the post‐LT survival model after excluding acetaminophen‐induced ALF, using the same covariates as the primary Cox model; and (2) we added MELD score, mechanical ventilation, life support (pressors), and hemodialysis (all at waitlist) as covariates in the primary Cox model.

## 3 Results

### 3.1 Overall Patient Cohort

Among 232 179 observations in the dataset from UNOS for LT, there were 7114 observations for listing as Status 1 between February 27, 2002 and June 30, 2023. The analysis dataset for WL outcomes included 6600 patients (Figure S1). Among these, 840 patients (13%) were listed for ALF after a weekend on Monday or after a holiday‐Monday weekend and are hereafter referred to as “listed after a weekend.” Baseline characteristics were comparable between patients listed after a weekend and on other days (Table 1). Among the listed patients, 3830 patients received LT (Table S1). The mean number of days on WL before LT was 2.8 (median 2, IQR 1, 3). Although MELD scores were similar between groups (approximately 35, *p* = 0.454), the weekend‐listed group had marginally higher proportions of patients receiving life support (49.0% vs. 45.1%, *p* = 0.11), on mechanical ventilation (55.8% vs. 53.0%, *p* = 0.20), and with dialysis initiated in the prior week (16.0% vs. 13.1%, *p* = 0.19).

**TABLE 1:** Comparison of demographic and clinical characteristics of patients waitlisted after a weekend or on other days for ALF as status 1 in the US between 2002 and 2023.

Variable	Listing after a weekend *n* = 840	Listing on other days *n* = 5760	*p*
Age, mean (SD)	42.3 (14.6)	42.7 (14.8)	0.71
Male (%)	316 (37.6)	2188 (38.0)	0.87
Race/ethnicity (%)			0.35
White	151 (61.3)	3489 (60.6)	
Black	132 (15.7)	1009 (17.5)	
Hispanic	114 (13.6)	718 (12.5)	
Asian	59 (7.0)	445 (7.7)	
Other	20 (2.4)	99 (1.7)	
Medicaid coverage (%)	148 (17.6)	1146 (19.9)	0.13
Acuity circle era (%)	81 (9.6)	631 (11.0)	0.28
ALF etiology acetaminophen toxicity^a^ (%)	141 (16.8)	952 (16.5)	0.89
MELD score at waitlist, median (IQR)	35 (29–41)	35 (29–41)	0.36
Ventilator at waitlist (%)	469 (55.8)	3053 (53.0)	0.20
Life support (vasopressors) at waitlist (%)	412 (49.0)	2598 (45.1)	0.11
On dialysis at waitlist	134 (16.0)	755 (13.1)	0.19
Transplant center volume^b^, mean (SD)	1635 (846)	1558 (799)	0.010

^a^ Compared to other ALF etiologies.

^b^ Total number of liver transplants of the center during the study period.

### 3.2 WL Outcomes

WL outcomes for the overall cohort consisted of LT (*n* = 3830, 58%), died or delisted due to clinical deterioration from WL (*n* = 1393, 21.1%), and SS (*n* = 1377, 20.9%). Compared to patients listed on other days, patients listed after a weekend had a lower rate of SS (17.3% listing after a weekend vs. 21.3% listing on other days) and a higher rate of LT (61.2% listing after a weekend vs. 57.6% listing on other days; *p* = 0.021; Table 2). In the adjusted multinomial regression model, listing after a weekend was associated with a lower likelihood of SS (RRR 0.74, 95% CI 0.60–0.90, LT as reference). After adjusting for potential confounding factors, WL mortality (with LT as reference) was not found to be associated with listing after a weekend (RRR 0.96, 95% CI: 0.80–1.15). The results of the multinomial models for each WL outcome are presented in Table 3. Of note, acetaminophen toxicity and AC era were associated with a higher likelihood of receiving LT.

**TABLE 2:** Comparison of patients waitlisted after a weekend or on other days for ALF.

WL outcome (%)	Listing after a weekend *n* = 840	Listing on other days *n* = 5760	*p*
LT	514 (61.2)	3316 (57.6)	0.021
WL mortality	181 (21.5)	1212 (21.0)	
SS	145 (17.3)	1232 (21.4)	

**TABLE 3:** Results of multivariable multinomial model for LT WL outcomes.

Variable	WL mortality		Spontaneous survival	
	RRR^a^	95% CI	RRR^a^	95% CI
Listing after a weekend	0.97	0.81–1.17	0.74	0.61–0.90
Male	0.96	0.85–1.10	1.12	0.98–1.27
Age, per year	1.01	1.00–1.01	0.99	0.99–0.99
Race/ethnicity				
White	—	—	—	—
Black	1.02	0.87–1.20	0.62	0.51–0.74
Hispanic	0.91	0.75–1.10	1.02	0.85–1.21
Asian	0.97	0.82–1.18	0.85	0.72–0.98
Other	0.96	0.94–0.98	0.72	0.71–0.73
Acetaminophen toxicity^b^	1.87	1.56–2.23	3.07	2.60–3.61
AC era	0.67	0.56–0.85	0.77	0.63–0.96
Medicare	1.12	0.97–1.33	1.03	0.88–1.22
Transplant center volume	1.00	1.00–1.00	1.00	1.00–1.00

*Note:* Models are adjusted for sex, age, ALF etiology, pre−/post‐AC era, insurance, primary payer (Medicaid vs. other), race/ethnicity, and transplant center volume.

Abbreviations: CI, confidence interval; RRR, relative risk ratio.

^a^ Reference outcome LT.

^b^ Compared to other ALF etiologies.

### 3.3 Post‐LT Patient Survival

Post‐LT survival at 1 year was evaluated in 3830 patients who underwent LT. All‐cause mortality at 1 year after LT was 17%. Among patients listed after a weekend, post‐LT mortality at 1 year was higher compared to those listed on other days (20.2% vs. 16.5%, respectively; log‐rank test, *p* = 0.04). Listing after a weekend was associated with higher post‐LT mortality in unadjusted (HR 1.25, 95% CI: 1.02–1.55) and adjusted Cox proportional hazards models (HR 1.25, 95% CI: 1.01–1.54; Table 4).

**TABLE 4:** Estimated effect of listing after a weekend on all‐cause mortality at 1‐year follow‐up for 3830 post‐LT patients using multivariable Cox proportional hazards model.

Model	HR	95% CI	*p*
Unadjusted	1.25	1.02–1.55	0.035
Adjusted	1.25	1.01–1.54	0.038
Weighted			
Model 1^a^	1.24	1.01–1.53	0.046
Model 2^b^	1.22	0.99–1.50	0.065

*Note:* Models are adjusted for sex, age, ALF etiology, pre−/post‐AC era, insurance, primary payer (Medicaid vs. other), race/ethnicity, and transplant center volume.

Abbreviations: CI, confidence interval; HR, hazard ratio.

^a^ Waitlist mortality was considered censored for weights.

^b^ Spontaneous survival was included as censored for weights.

In the logistic regression models for estimation of the probability of censoring before LT, the direction of the estimated odds ratios was different for WL mortality and SS: the covariates of AC era and ALF etiology were associated with WL mortality, whereas WL after a weekend, White race/ethnicity, AC era, ALF etiology, and age were associated with SS (Table S2). These logistic model estimates were congruous with the multivariate multinomial regression estimates for WL outcomes. After applying IPW for spontaneous survival, covariates were balanced between groups (standardized mean differences < 0.1). When censoring for SS was accounted for with IPW, the significant mortality difference persisted with HR of 1.24 (95% CI 1.00–1.53). When censoring for WL mortality was accounted for in model 2, the estimate for HR was similar and did not reach the considered significance threshold (HR 1.22, 95% CI 0.99–1.50).

In additional sensitivity analyses, a model excluding acetaminophen‐induced ALF showed that weekend listing remained associated with higher post‐LT mortality (HR 1.33, 95% CI 1.07–1.64), with similar effects of other covariates to the main model. Also, adding MELD score, mechanical ventilation, life support (pressors), and hemodialysis as covariates to the primary Cox model yielded a comparable result (HR 1.24, 95% CI 1.001–1.52).

## 4 Discussion

This study found that in ALF patients, listing after a weekend was associated with lower rates of spontaneous recovery and higher rates of LT. In patients who underwent LT, listing after a weekend was associated with higher post‐LT mortality at 1 year. Accounting for censoring bias using IPW for SS or WL mortality as drop‐out before LT in two separate analyses, we found essentially similar higher post‐LT mortality in patients listed after a weekend, although the confidence interval of the hazard ratio did not reach the significance threshold for the IPW group with SS drop‐out. To the best of our knowledge, this is the first study to investigate the impact of delayed listing (as indicated by listing after a weekend) on post‐LT outcomes specifically in ALF. While previous studies have examined weekend effects on organ acceptance, transplant center activities, and other aspects of transplantation workflow such as offer decline rates over the weekend [13, 14], our work uniquely focuses on how delayed listing practices affect clinical outcomes in this urgent transplant indication.

Lower rates of SS on WL and higher post‐LT mortality associated with listing after a weekend suggest deteriorated clinical condition at listing for these patients. The clinical deterioration may be linked to delays in LT evaluation over weekends. Other factors associated with lower SS rates included increasing age, Black race, and AC era, consistent with previous observations [11, 16, 17]. Our results are also consistent with higher WL mortality and SS in ALF due to acetaminophen toxicity. In other words, it is suggested that patients listed for ALF due to acetaminophen toxicity might have been at higher risk of death before LT (likely with more severe form of disease) but also had a higher chance of spontaneous recovery if the condition was less severe [11, 17]. Although differences in weekend and weekday admissions can possibly affect WL outcomes in ALF patients with acetaminophen toxicity, we did not find differences in rates of acetaminophen toxicity etiology according to listing day.

Previous studies on the quality of care on weekends are primarily motivated by concerns that the lack of timely care on weekends may affect outcomes. Timely care is particularly crucial in acute clinical conditions with immediate high mortality rates, such as neurologic, cardiac, or surgical emergencies [1, 23]. The weekend effect in LT outcomes was evaluated in LT surgeries on weekends compared to weekdays in two prior studies with the European transplant database and UNOS [5, 7]. These studies have not consistently shown a weekend effect in LT outcomes; the variation in outcomes was attributed to the presence of senior staffing availability perioperatively, regardless of business hours. Furthermore, contrasting other LT indications, ALF admissions require immediate consideration for listing in coordination with multidisciplinary discussions. Limitations in multidisciplinary team discussions and the limited availability of senior physicians, experts, and allied health professionals on weekends may be a factor in our results. Other potential factors include delays in administrative processes, including insurance authorization. We included Medicaid coverage in our adjusted analyses; however, these adjustments did not meaningfully change estimates.

A causality of an observed weekend effect can be more precisely estimated when treatment timeliness directly influences outcomes and variations between weekend and weekday hospitalizations are minimal or negligible [8]. ALF care offers an ideal model to study the weekend effect on LT outcomes for several reasons: ALF and listing practices in LT patients provide a naturally controlled environment as an elective LT for ALF is unlikely; admission day can be considered random with minimal selection bias because all ALF patients will be admitted regardless of the day of the week due to their critical condition; and time from WL to LT is fairly uniform in the current US LT allocation system. While this study's scope might appear limited given that ALF comprises only approximately 3% of overall LT volume, insights gained from examining the workflow of this urgent transplant indication may yield valuable system‐wide improvements applicable to broader transplantation practices.

Our observed associations may reflect delays in clinical and administrative listing practices on weekends, resulting in a more deteriorated clinical condition by the time of the listing. However, the underlying differences in the clinical status of weekend vs. weekday patients could not be ascertained. A possibility exists that disparate clinical outcomes attributed to the weekend effect stem from health status differences between weekend and weekday ALF admissions. For example, early evidence of the weekend effect from studies showing higher perinatal mortality in weekend births was partially attributed to higher acuity weekend cases due to weekday elective inductions [24, 25]. Similarly, in ALF, patients who recover over the weekend then were not listed and not captured in our dataset, while those still needing listing after the weekend were in a more deteriorated condition at listing. This selection bias may explain the observed lower SS in our analyses. Therefore, future study designs should include ALF patients who were not listed, examine admission day effects on WL evaluation duration, and assess staffing variations to explore specific pathways underlying our results. If weekend‐related disruptions exist in the liver transplantation evaluation and treatment workflow, specific delay mechanisms warrant investigation. Weekend staffing variations and expertise differences may contribute to the observed weekend effect, supported by previous studies showing transplant center volume influence on waitlist outcomes in ALF from the UNOS database and living donor transplantation cohort analyses [16, 26]. In our current study, the weekend effect persisted despite adjusting our models for transplant center volume, contrasting with a prior UNOS dataset study that demonstrated transplant center volume influences waitlist outcomes in ALF [16]. Notably, center volume showed no association with ALF outcomes in our multivariable analyses, which challenges the hypothesis that delays stem from limited healthcare resource availability. This negative finding suggests that specific center‐level operational factors, rather than simply transplant volume, may drive the observed disparity. Additionally, Medicaid insurance status did not affect outcomes, suggesting that potential insurance approval delays are not specific to certain payer types, including Medicaid.

The strength of our current study lies in the fact that this is the first study to assess the weekend effect on WL outcomes and post‐LT mortality in ALF. We have utilized a large‐scale national transplant database with longitudinal data. We used robust causal methodology to minimize confounding. In the LT analyses, we addressed censoring bias using IPW. The causal graph that guided our analysis relies on our assessment of the consequences of steps involved in admission to LT of ALF patients and can be expanded with more details. Nevertheless, this study has several limitations mainly due to reliance on retrospective, registry‐based data. There was no information on admission dates for patients who had not been transplanted. The UNOS/OPTN registry lacks the granularity to address this. This information would have provided direct evidence of potential delays in listing evaluations on the weekends. Critical information on ALF patients who were not listed, such as the date of admission, was not present in the UNOS dataset. Etiology categorization in the UNOS database was not sufficiently granular to explore the impact of other ALF etiologies on clinical outcomes according to the listing day. This drop‐out before listing is potentially more frequent during weekends and may result in selection bias. Our findings based on the national US database may have limited generalizability to other healthcare systems and in various regions within the US due to the region‐to‐region variation in transplant practices.

In conclusion, patients with acute liver failure (ALF) who are listed for liver transplantation after a weekend demonstrate lower rates of spontaneous recovery and higher post‐transplant mortality. Although actual delays were not directly quantified in this study, these disparities may reflect delays in evaluation and listing processes associated with reduced availability of clinical resources during weekends. Our findings highlight the need to examine operational factors within hospital systems that may contribute to delays in time‐sensitive transplant evaluations. Future research should assess whether these patterns persist across diverse healthcare settings and incorporate data on all ALF admissions to better define the impact of weekend‐related workflow on transplant outcomes.

## Conflicts of Interest

The authors declare no conflicts of interest.

## Supporting information


**Data S1:** Supporting Information.

## Data Availability

The data that support the findings of this study are available on request from the corresponding author. The data are not publicly available due to privacy or ethical restrictions.
