# Supplementary material for: The Impact of Waitlisting After a Weekend on Transplant‐Related Outcomes for Patients With Acute Liver Failure in the US
Source: JGH Open. 2025 Sep 1;9(9):e70271. doi: 10.1002/jgh3.70271 (PMC12400070; doi:10.1002/jgh3.70271)

**Supplemental Table 1.**

Comparison of characteristics and LT outcomes of LT patients listed after a weekend or on other days for ALF as Status 1 in the U.S. between 2002 and 2023.

| Variable | Listing after a weekend  n=514 | Listing on other days  n=3316 | *P*-value |
| --- | --- | --- | --- |
| Age, mean (SD) | 43.1 (14.2) | 43.4 (14.7) | 0.717 |
| Male (%) | 194 (37.7) | 1290 (38.9) | 0.650 |
| MELD score at WL entry, median (IQR) | 35.5 (30-41) | 35 (29-41) | 0.472 |
| Days from admission to listing, mean (SD) | 4.4 (8) | 5 (64) | 0.843 |
| Days on WL, median (IQR) | 2 (1-3) | 2 (1-3) | 0.091 |
| Cold ischemia time, hours, mean (SD) | 6.5 (4.2) | 6.4 (2.9) | 0.404 |
| Race/ethnicity (%) |  |  | 0.204 |
| White | 300 (58.4) | 1935 (58.4) |  |
| Black | 91 (17.7) | 630 (19.0) |  |
| Hispanic | 77 (15.0) | 413 (12.5) |  |
| Asian | 33 (6.4) | 278 (8.4) |  |
| Other | 13 (2.5) | 60 (10.9) |  |
| Medicaid coverage (%) | 94 (18.3) | 636 (19.2) | 0.675 |
| Acuity circle era (%) | 54 (10.5) | 418 (12.6) | 0.202 |
| ALF etiology acetaminophen toxicity (%) | 60 (11.7) | 361 (10.9) | 0.649 |
| Transplant center volume, mean (SD) | 1666 (854) | 1589 (809) | 0.048 |
| LT mortality at 1 year (%) | 104 (20.1) | 547 (16.5) | 0.042 |

**Supplemental Table 2.**

Characteristics associated with censoring; logistic regression estimates included in inverse probability weighting procedures.

|  | **Waitlist mortality** | | | **Spontaneous survival** | | |
| --- | --- | --- | --- | --- | --- | --- |
| **Variable** | **Estimate** | **Standard error** | ***P*-value** | **Estimate** | **Standard error** | ***P*-value** |
| (Intercept) | -1.2123 | 0.2771 | < 0.001 | -0.1661 | 0.269 | 0.537 |
| Listed after a weekend (1) | -0.038 | 0.0934 | 0.684 | -0.3027 | 0.1031 | 0.003 |
| Sex (Male) | -0.0408 | 0.0664 | 0.539 | 0.1049 | 0.0688 | 0.127 |
| Race/ethnicity | | | | | | |
| Black | 0.03 | 0.0853 | 0.725 | -0.4751 | 0.0991 | < 0.001 |
| Hispanic | -0.083 | 0.1023 | 0.417 | 0.0319 | 0.099 | 0.747 |
| Asian | -0.0201 | 0.1213 | 0.868 | -0.1834 | 0.1306 | 0.16 |
| Other | -0.0188 | 0.2378 | 0.937 | -0.3259 | 0.2588 | 0.208 |
| Age | -0.0001 | 0.0131 | 0.992 | -0.0393 | 0.0133 | 0.003 |
| Age^2 | 0.0001 | 0.0001 | 0.613 | 0.0003 | 0.0002 | 0.028 |
| Acetaminophen toxicity | 0.624 | 0.0917 | < 0.001 | 1.12 | 0.0841 | < 0.001 |
| AC era | -0.3788 | 0.1087 | < 0.001 | -0.271 | 0.1111 | 0.015 |
| Medicare | 0.1286 | 0.0816 | 0.115 | 0.0286 | 0.0838 | 0.733 |

^a^ Compared to White

b Compared to other ALF etiologies

**Supplemental Figure 1.**

Flow diagram of analysis dataset.

Liver transplant patients in UNOS dataset

February 27, 2002 to June 30, 2023

N=232 179

Acute liver failure, n=7114

Exclusion criteria

Re-transplantation, n=300

Waitlist outcome not specified, n=165

Waitlist days >14, n=49

Waitlist analysis cohort, n=6600

Listing after weekend, n = 840

Listing on other days, n = 5860

Liver transplantation, n=3830

Listing after weekend, n = 514 Listing on other days, n = 3316

Waitlist mortality, n=1393

Listing after weekend, n = 181 Listing on other days, n = 1212

Spontaneous survival, n=1377

Listing after weekend, n = 145 Listing on other days, n = 1232

**Supplemental Figure 2.**

Direct acyclic graph used for analysis of the weekend effect on WL and LT outcomes.


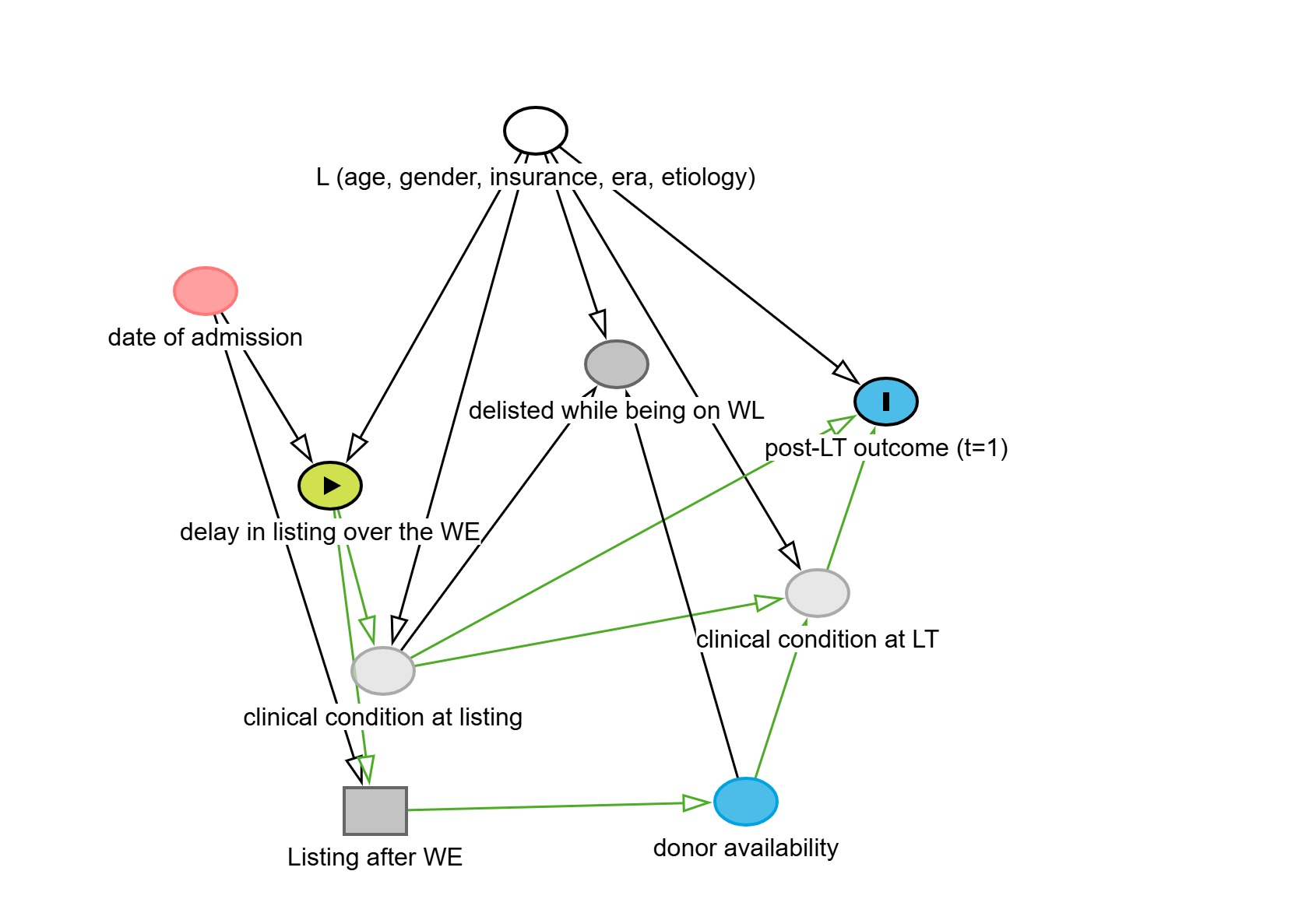

Supplement: Supplementary file 1 — Data S1: Supporting Information. [file JGH3-9-e70271-s001.docx]
